# ﻿Two new species of the genus *Malaxa* Melichar, 1914 from China (Hemiptera, Fulgoromorpha, Delphacidae, Tropidocephalini)

**DOI:** 10.3897/zookeys.1229.143177

**Published:** 2025-02-28

**Authors:** Sha-Sha Lv, Hong-Xing Li, Lin Yang, Yu-Bo Zhang, Xiang-Sheng Chen

**Affiliations:** 1 Institute of Entomology, Guizhou University, Guiyang, Guizhou, 550025, China; 2 The Provincial Special Key Laboratory for Development and Utilization of Insect Resources of Guizhou, Guizhou University, Guiyang, Guizhou, 550025, China; 3 Guizhou Light Industry Technical College, Guiyang, Guizhou, 561113, China; 4 College Agriculture, Anshun University, Anshun, Guizhou, 561000, China

**Keywords:** Bamboo host, checklist, Fulgoromorpha, identification key, Oriental region, planthopper, taxonomy, Tropidocephalini

## Abstract

Two new species of the genus *Malaxa* Melichar, 1914 from Southwest China, *M.chongzuoensis* Lv & Chen, **sp. nov.** from Guangxi Zhuang Autonomous Region, and *M.longispina* Lv & Chen, **sp. nov.** from Guangdong Province, are described and illustrated. These bring the total number of species in the genus to 13, with nine recorded from China. A checklist and map of all known species of *Malaxa* are provided, together with an identification key for Chinese species.

## ﻿Introduction

Melichar (1914) established the delphacid planthopper genus *Malaxa* with the type species *M.acutipennis* Melichar, 1914 from the Philippines and placed this genus in the tribe Tropidocephalini of the subfamily Delphacinae (Hemiptera: Fulgoromorpha: Delphacidae) (Muir 1915, 1916). Until now, 11 species have been recorded in the genus (Bartlett and Kennedy 2018; Li et al. 2019; Bourgoin 2024). In turn, Muir (1919, 1926) described four new species, *M.javanensis* Muir, 1919 and *M.bispinata* Muir, 1926 from Indonesia, *M.nigra* Muir, 1919 from Philippines, and *M.obtusipennis* Muir, 1919 from Malaysia. Ding et al. (1986) and Yang and Yang (1986) added three new species from China, *M.delicata* Ding & Yang, 1986, *M.fusca* Yang & Yang, 1986 and *M.semifusca* Yang & Yang, 1986. Chen et al. (2006) and Hou et al. (2013) both reviewed the Chinese species of *Malaxa*, and added a new species, *M.hunanensis* Chen, 2006. Bartlett and Kennedy (2018) reviewed the New World species attributed to the genus, redescribed the type species *M.acutipennis* Melichar, 1914, and transferred *M.occidentalis* Muir, 1926 and *M.gracilis* Fennah, 1945 to *Lamaxa* Bartlett & Kennedy, 2018, and *M.microstyla* Muir, 1930 to *Xalama* Bartlett & Kennedy, 2018. Li et al. (2019) described two new species, *M.hamuliferum* Li, Yang & Chen, 2019 and *M.tricuspis* Li, Yang & Chen, 2019 from China. This genus is known to occur in the Oriental region, with seven species in China, two species in the Philippines, two species in Indonesia, and one species in Malaysia (Melichar 1914; Muir 1919, 1926; Ding et al. 1986; Yang and Yang 1986; Chen et al. 2006; Ding 2006; Hou et al. 2013; Li et al. 2019; Bourgoin 2024).

Here we describe and illustrate two new species of *Malaxa* discovered in southern China, and provide a checklist of species, a distribution map, and an updated key to Chinese species.

## ﻿Material and methods

The external morphology terminologies follow Bourgoin (1987) for male genitalia and Bourgoin et al. (2015) for wing venation. The zoogeographic regionalization scheme follows Holt et al. (2013). Specimens were collected by sweeping. Dry male specimens were used for the descriptions and illustrations. Body length was measured from the apex of the vertex to the tip of the forewings. All measurements are in millimeters (mm). Color pictures for adult habitus were taken using the KEYENCE VHX-6000 system. External morphology and drawings were done with a Leica MZ 12.5 stereomicroscope. The photographs and illustrations were scanned with a CanoScan LiDE 200 and imported into Adobe Photoshop v. 6.0 for labeling and plate composition. The genital segments of the examined specimens were macerated in 10% NaOH, and then transferred to glycerol in small plastic tubes pinned together with the specimens for examination. The distribution map was generated with ArcGIS v. 10.7.

The type specimens examined are deposited in the Institute of Entomology, Guizhou University, Guiyang, Guizhou Province, China (IEGU).

## ﻿Taxonomy

### ﻿ Malaxa

Taxon classificationAnimaliaHemipteraDelphacidae

Melichar, 1914

C0792920-7DFF-5D89-B47E-5781E963BDF7

Malaxa Melichar, 1914: 275; Muir 1926: 7; Metcalf 1943: 103; Fennah 1945: 429; Yang and Yang 1986: 56; Ding et al. 1986: 418, 1999: 443; Chen et al. 2006: 160; Ding 2006: 150; Bartlett 2009: 387; Hou et al. 2013: 866; Bartlett and Kennedy 2018: 514; Li et al. 2019: 44.

#### Type species.

*Malaxaacutipennis* Melichar, 1914, original designation.

**Diagnosis.** For the diagnosis of *Malaxa* see Hou et al. (2013: 866) and Li et al. (2019: 44).

#### Host plants.

Bamboo (Poales: Poaceae: Bambusoideae).

#### Distribution.

**Figure 1.**
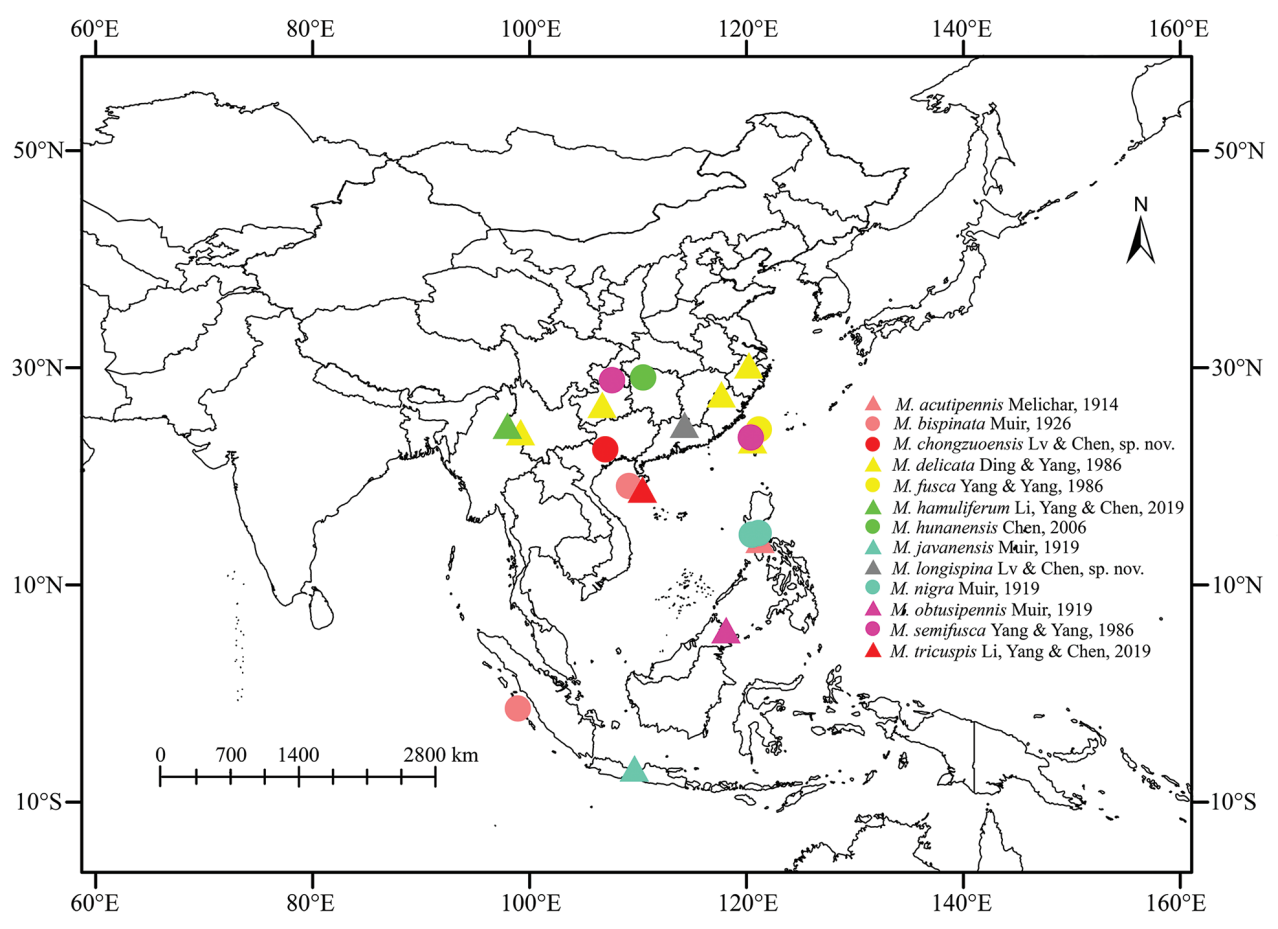
Geographic distributions of species of *Malaxa* Melichar, 1914.

China, Indonesia, Malaysia, Philippines (Fig. 1).

##### ﻿Checklist and distributions of species of *Malaxa* Melichar, 1914

*M.acutipennis* Melichar, 1914; Philippines (Luzón)

*M.bispinata* Muir, 1926; Indonesia (Mentawai), China (Hainan)

*M.chongzuoensis* Lv & Chen, sp. nov.; China (Guangxi)

*M.delicata* Ding & Yang, 1986; China (Fujian, Guizhou, Taiwan, Yunnan, Zhejiang)

*M.fusca* Yang & Yang, 1986; China (Taiwan)

*M.hamuliferum* Li, Yang & Chen, 2019; China (Yunnan)

*M.hunanensis* Chen, 2006; China (Hunan)

*M.javanensis* Muir, 1919; Indonesia (Java)

*M.longispina* Lv & Chen, sp. nov.; China (Guangdong)

*M.nigra* Muir, 1919; Philippines (Luzón)

*M.obtusipennis* Muir, 1919; Malaysia (Borneo)

*M.semifusca* Yang & Yang, 1986; China (Guizhou, Taiwan)

*M.tricuspis* Li, Yang & Chen, 2019; China (Hainan)

### ﻿Key to Chinese species of *Malaxa* Melichar, 1914

(modified from Li et al. 2019)

**Table:** 

1	Postclypeus yellow; forewings with apical veins CuA and MP_3_ diverging apically, posterior half of apical forewings dark brown (Chen et al. 2006: figs 2, 3)	***M.semifusca* Yang & Yang, 1986**
–	Postclypeus with basal half blackish-brown; forewings with apical veins CuA and MP_3_ fused, first and second apical cells hyaline	**2**
2	Anal segment without process; aedeagus with phallobase	**3**
–	Anal segment with a long process; aedeagus without phallobase	**6**
3	Genae all dark brown; forewings with basal half most yellow, apical half most dark brown	**4**
–	Genae dark brown in part; forewings mostly hyaline	**5**
4	Forewings (Fig. 7) with basal 1/2 bearing a reverse hyaline V-shaped marking, fifth apical cell dark brown in part; dorsal margin of phallobase (Fig. 11) with a slender horned process	***M.chongzuoensis* Lv & Chen, sp. nov.**
–	Forewings with basal 1/2 bearing a hyaline V-shaped marking, fifth apical cell dark brown; dorsal margin of phallobase without a slender horned process (Li et al. 2019: figs 8, 12)	***M.hamuliferum* Li, Yang & Chen, 2019**
5	Vertex with apical half and basal half of frons dark brown; middle part of apical half of forewings without grayish-brown markings; pygofer with three medioventral processes very distinct (Hou et al. 2013: figs 1–3, 8)	***M.bispinata* Muir, 1926**
–	Vertex and frons (Figs 16, 18) grayish-white; middle part of apical half of forewings (Fig. 17) with grayish-brown markings; pygofer (Figs 19, 20) with two medioventral processes not distinct	***M.longispina* Lv & Chen, sp. nov.**
6	Genae dark brown; in posterior view, process of anal segment situated in middle of ventral margin (Chen et al. 2006: figs 21, 23, 25)	***M.hunanensis* Chen, 2006**
–	Genae mostly dark brown but apical with small part yellow; in posterior view, process of anal segment situated on left side of ventral margin	**7**
7	Gonostyles with apex not forked; aedeagus with three processes (Li et al. 2019: figs 22–24)	***M.tricuspis* Li, Yang & Chen, 2019**
–	Gonostyles with apex forked; aedeagus with two processes	**8**
8	Area between lateral carinae of pronotum dark brown; two branches of outer apical angle of gonostyles subequal; aedeagus with a small spine situated near basal third, directed caudally (Chen et al. 2006: figs 30, 37, 38)	***M.delicata* Ding & Yang, 1986**
–	Area between lateral carinae of pronotum mostly yellow; two branches of outer apical angle of gonostyles unequal; aedeagus with a small tooth situated near middle, directed right (Chen et al. 2006: figs 11, 18, 19)	***M.fusca* Yang & Yang, 1986**

### ﻿ Malaxa chongzuoensis

Taxon classificationAnimaliaHemipteraDelphacidae

Lv & Chen sp. nov.

498C4CA5-B9D9-5C0C-A6D0-55AF901A5E41

https://zoobank.org/BB6FCADA-56F5-4854-94F3-16AA5FA5F82E

Figs 2 , 3 , 6–15 , 26

#### Type material.

***Holotype***: China • ♂; Guangxi Zhuang Autonomous Region, Chongzuo City, Longzhou County, Zhubu Township, Nonggang Village; 22°39'N, 106°57'E; sweeping, 16 August 2024; Sha-Sha Lv and Xiang-Sheng Chen leg.; IEGU. ***Paratypes***: China • 1♂, 4♀♀; same collection data as for holotype; IEGU.

#### Diagnosis.

**Figures 2–5.**
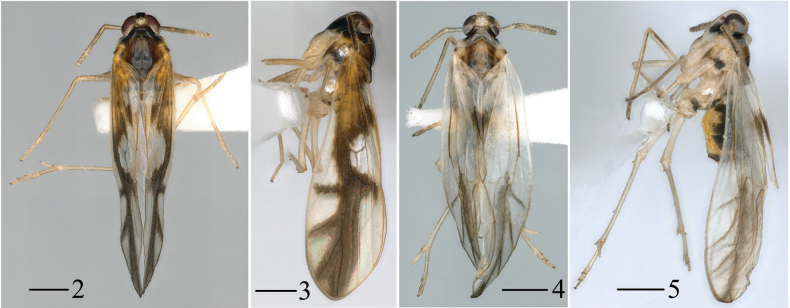
*Malaxachongzuoensis* Lv & Chen, sp. nov., male **2** habitus, dorsal view **3** habitus, lateral view **4, 5***Malaxalongispina* Lv & Chen, sp. nov., male **4** habitus, dorsal view **5** habitus, lateral view. Scale bars: 0.5 mm (**2–5**).

**Figures 6–15.**
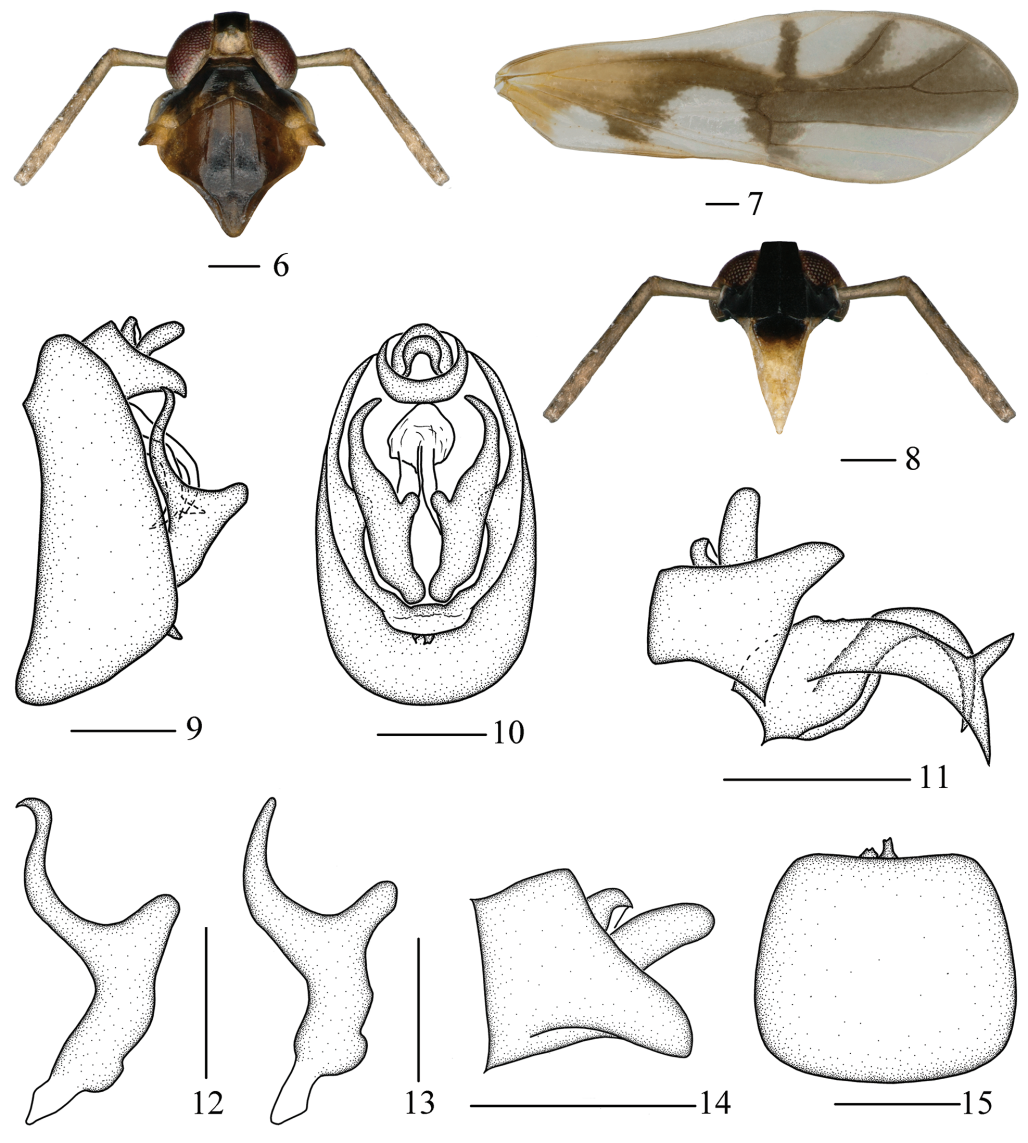
*Malaxachongzuoensis* Lv & Chen, sp. nov., male **6** head and thorax, dorsal view **7** forewing **8** frons, ventral view **9** male genitalia, lateral view **10** male genitalia, posterior view **11** anal segment and aedeagus, lateral view **12** gonostyle, lateral view **13** gonostyle, posterior view **14** anal segment, lateral view **15** pygofer, ventral view. Scale bars: 0.2 mm (**6–15**).

The salient features of the new species include: vertex (Figs 2, 6) with apical half brownish-black; frons and genae (Figs 3, 8) black; mesonotum (Figs 2, 6) blackish-brown at middle, rest tawny to reddish-brown; forewings (Fig. 7) with a reversed hyaline V-shaped marking; pygofer (Fig. 15) in ventral view medioventral processes asymmetrical; outer process of gonostyles (Fig. 12) snakelike in lateral view; dorsal margin of phallobase (Fig. 11) with a slender horned process at apical 1/3.

#### Description.

***Measurements*.** Total length: male 3.5–3.7 mm (*N* = 2), female 4.1–4.5 mm (*N* = 4).

***Coloration*.** General color pale yellowish-brown (Figs 2, 3). Vertex (Figs 2, 6) with apical half brownish-black, basal half yellowish-brown. Frons (Fig. 8) and genae (Figs 3, 8) black. Clypeus (Fig. 8) with basal half brownish-black. Eyes (Figs 6, 8) reddish-brown. Pronotum (Figs 2, 6) brown to black except lateral sides yellow. Mesonotum (Figs 2, 6) blackish-brown at middle, rest tawny to reddish-brown. Outer part of tegulae (Figs 2, 6) black brown, inner part yellowish-white. Forewings (Fig. 7) greyish-white, hyaline, veins gray to light yellowish-brown, basal 1/4 light yellowish-brown except areas around bifurcation of Pcu and A1, basal 1/4 to middle part with an arched dark brown stripe, forming a reverse hyaline V-shaped marking, along ScP, ir, RP and area between MP_1_ and MP_2_ dark brown.

***Head and thorax*.** Vertex (Fig. 6) slightly longer than wide at base (1.07: 1), width at apex narrower than at base (0.83: 1), submedian carinae uniting slightly beyond middle, apex produced in front of eyes, apical margin straight, greatest length of basal compartment shorter than wide at base of vertex (0.67: 1). Frons (Fig. 8) longer in middle line than wide at widest portion (about 1.28: 1), widest at apex, median carina simple. Postclypeus (Fig. 8) wide at base as wide as frons at apex. Antennae (Figs 6, 8) very long, cylindrical, surpassing apex of clypeus, scape longer than wide, shorter than pedicel (0.46: 1). Pronotum (Fig. 6) with lateral carinae not attaining hind margin, longer than vertex in midline (0.75: 1). Mesonotum (Fig. 6) with lateral carinae not attaining hind margin, longer than 1.67 times pronotum and vertex combined. Forewings (Fig. 7) slender, longer than maximal width (3.02: 1).

***Male genitalia*.** Pygofer in lateral view (Fig. 9) ventral margin longer than dorsal margin, in posterior view (Fig. 10) with opening longer than wide, in ventral view (Fig. 15) medioventral processes asymmetrical, concave medially, left process shorter. Gonostyles (Figs 12, 13) long, in lateral view apical half bifurcated into two processes, outer process slender and curved, snakelike, tapering to apex, inner process short and thick, rounded at apex; in posterior view C-shaped. Male genitalia (Fig. 11) with phallobase, aedeagus tubular, vaulted ventrally, tapering to apex; phallobase wide, curved ventrally, tapering to apex, dorsal margin with a slender horned process at apical 1/3. Anal segment (Figs 9, 14) small, ring-like.

#### Host plant.

*Indocalamustessellatus* (Munro) P. C. Keng (Poales: Poaceae: Bambusoideae) (Fig. 26).

#### Distribution.

China (Guangxi) (Fig. 1).

#### Etymology.

The new species is named after the city in which it was collected (Chongzuo) with the Latin adjectival suffix ‘-*ensis*’ meaning ‘from’.

#### Remarks.

This species (Figs 6–15) is similar to *M.hamuliferum* Li, Yang & Chen, 2019 (Li et al. 2019: figs 5–14), but differs from the latter in: (1) forewings with basal 1/2 bearing a reversed hyaline V-shaped marking (forewings with basal 1/2 bearing a hyaline V-shaped marking in *M.hamuliferum*); (2) dorsal margin of phallobase with a long horned process (dorsal margin of phallobase without a long horned process in *M.hamuliferum*); and (3) aedeagus shorter than phallobase (aedeagus longer than phallobase in *M.hamuliferum*).

### ﻿ Malaxa longispina

Taxon classificationAnimaliaHemipteraDelphacidae

Lv & Chen sp. nov.

EB7B8BB8-4C98-5F87-97A6-21FA6C929CCD

https://zoobank.org/5CB9A6AB-56C4-4064-A999-3086A7DC9918

Figs 4 , 5 , 16–25

#### Type materials.

***Holotype***: China • ♂: Guangdong Province, Shixing County, Luoba Town, Dashui Village; 24°46'N, 114°17'E; sweeping, 18 June 2023; Sha-Sha Lv leg.; IEGU. ***Paratypes***: China • 1♂, 3♀♀; Guangdong Province, Shixing County, Luoba Town, Dashui Village; 24°46'N, 114°17'E; sweeping, 18 June 2023; Sha-Sha Lv, Feng-E Li and Yong-Jin Sui leg.; IEGU.

#### Diagnosis.

**Figures 16–25.**
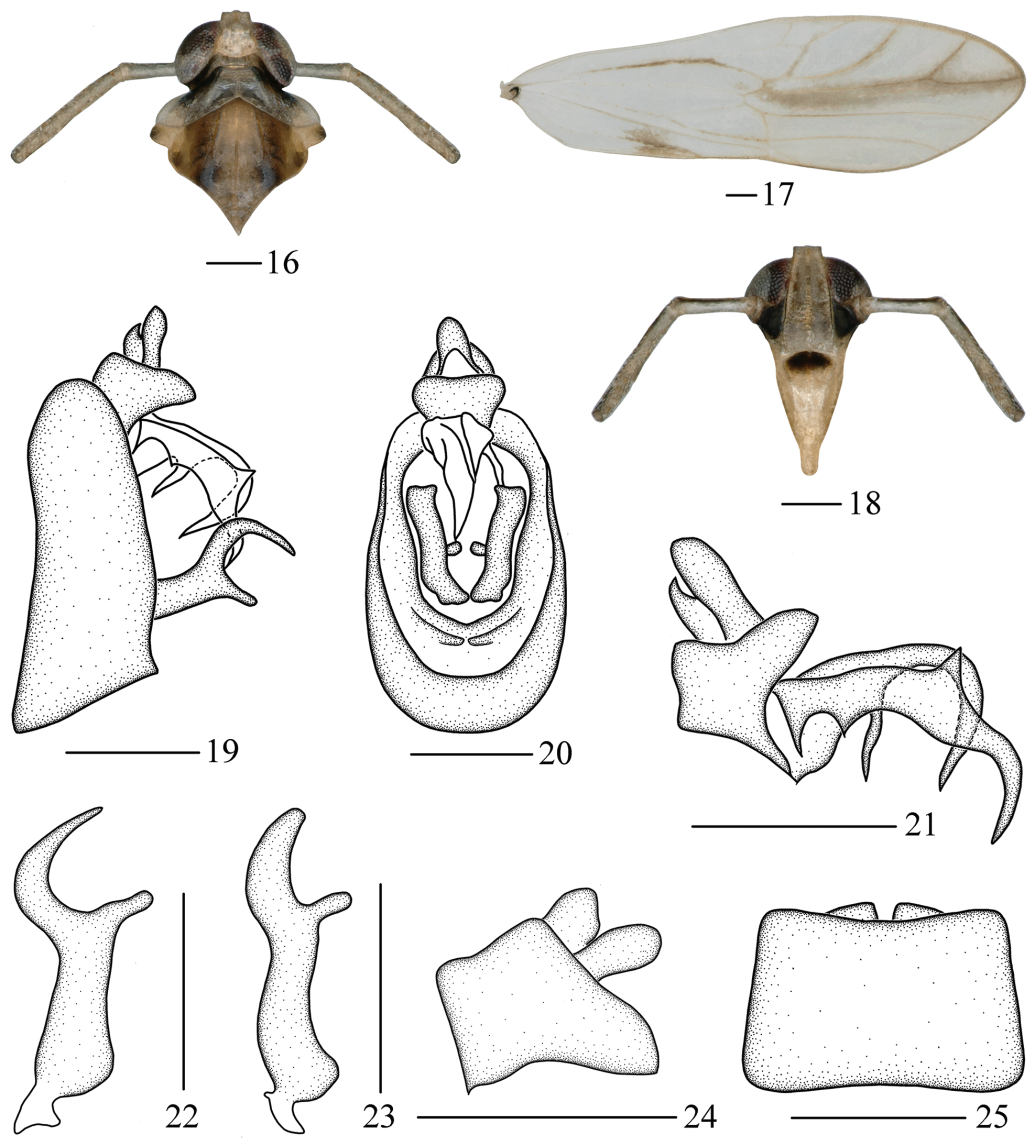
*Malaxalongispina* Lv & Chen, sp. nov., male **16** head and thorax, dorsal view **17** forewing **18** frons, ventral view **19** male genitalia, lateral view **20** male genitalia, posterior view **21** anal segment and aedeagus, lateral view **22** gonostyle, lateral view **23** gonostyle, posterior view **24** anal segment, lateral view **25** pygofer, ventral view. Scale bars: 0.2 mm (**16–25**).

The salient features of the new species include: vertex (Figs 4, 16) and frons (Fig. 18) light grayish-yellow; genae (Figs 5, 18) with basal 3/4 black markings; pronotum (Figs 4, 16) with outer sides of lateral carinae with black markings; forewings (Fig. 17) around ScP+R, ir, RP and MP_1_ with light greyish-brown marking; medioventral process of pygofer (Fig. 25) nearly rectangular in ventral view; gonostyles (Fig. 22) with apical half forming a C-shaped in lateral view; aedeagus (Fig. 21) narrows sharply near the middle, with a slender process.

#### Description.

***Measurements*.** Total length: male 3.3–3.4 mm (*N* = 2), female 4.1–4.3 mm (*N* = 3).

***Coloration*.** General color greyish-white to yellowish-brown (Figs 4, 5). Vertex (Figs 4, 16) and frons (Fig. 18) light grayish-yellow, frons with two indistinct light brown stripes. Genae (Figs 5, 18) with basal 3/4 black markings. Clypeus (Fig. 18) black at base, and rest light yellowish-brown. Eyes (Figs 16, 18) gray to reddish-black. Pronotum (Figs 4, 16) light grayish-brown, outer sides of lateral carinae with black markings. Mesonotum (Figs 4, 16) yellowish-brown, outer sides of lateral carinae with brown to black markings. Apex of tegulae (Figs 4, 16) black brown, rest light yellowish-brown. Forewings (Fig. 17) greyish-white, hyaline, veins gray to brown, areas after bifurcation of Pcu and A1 with yellowish-brown spot, around ScP+R, ir, RP and MP_1_ with light greyish-brown marking.

***Head and thorax*.** Vertex (Fig. 16) shorter submedially than wide at base (0.96: 1), width at apex narrower than at base (0.62: 1), submedian carinae uniting slightly beyond middle, apex produced in front of eyes, apical margin straight, greatest length of basal compartment shorter than wide at base of vertex (0.59: 1). Frons (Fig. 18) longer in middle line than wide at widest portion (about 2.17: 1), widest at apex, median carina simple. Postclypeus (Fig. 18) wide at base as wide as frons at apex. Antennae (Figs 16, 18) very long, cylindrical, surpassing apex of clypeus, scape longer than wide, shorter than pedicel (0.55: 1). Pronotum (Fig. 16) with lateral carinae not attaining hind margin, longer than vertex in midline (0.71: 1). Mesonotum (Fig. 16) with lateral carinae not attaining hind margin, longer than 1.61 times pronotum and vertex combined. Forewings (Fig. 17) slender, longer than maximal width (3.63: 1).

***Male genitalia*.** Pygofer in lateral view (Fig. 19) ventral margin longer than dorsal margin, ventral angles slightly produced, in posterior view (Fig. 20) with opening longer than wide, in ventral view (Fig. 25) medioventral processes nearly rectangular, concave medially. Gonostyles (Figs 22, 23) moderately long, in lateral view apical half bifurcated into two processes, forming a C-shaped, outer process long and bent, tapering to apex, inner process short, relatively rounded at apex; in posterior view curved, basal angles produced, outer process thick. Male genitalia (Fig. 21) with phallobase, aedeagus tubular, vaulted ventrally, basal part broad, narrows sharply near the middle, with a slender spinous process; phallobase long, curved ventrally, tapering to apex, basal part with two spiniform processes, dorsal margin with an angular process medially. Anal segment (Figs 19, 24) small, ring-like.

**Figure 26.**
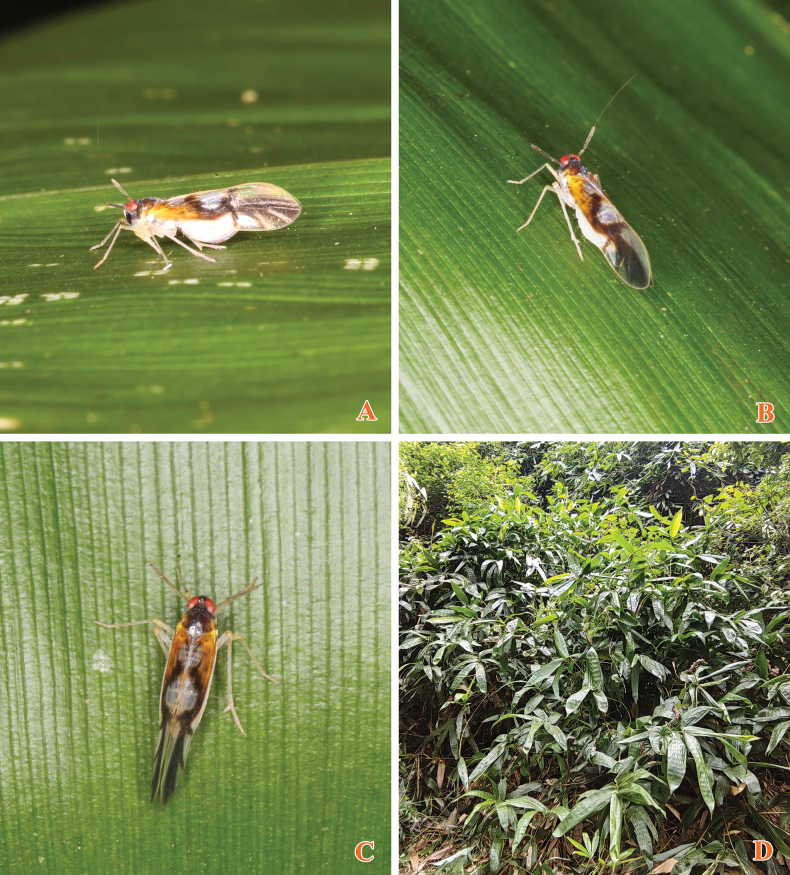
Adult of *Malaxachongzuoensis* Lv & Chen, sp. nov. resting on the leaf of *Indocalamustessellatus* (Munro) P. C. Keng (Poales: Poaceae: Bambusoideae). Photographed by Xiang-Sheng Chen.

#### Host plant.

*Indocalamus* sp. (Poales: Poaceae: Bambusoideae).

#### Distribution.

China (Guangdong) (Fig. 1).

#### Etymology.

The species name is a combination of the Latin word “*long*-” and “*spina*” (with the connecting letter “*i*”, i.e., “long spine”), referring to the ventral margin of the aedeagus with a long spinous process medially. The name is intended to be feminine.

#### Remarks.

This species (Figs 16–25) is similar to *M.bispinata* Muir, 1926 (Hou et al. 2013: figs 1–14), but differs from the latter in: (1) vertex and frons grayish-white (vertex with apical half and basal half of frons dark brown in *M.bispinata*); (2) middle part of apical half of forewings with grayish-brown markings (middle part of apical half of forewings without grayish-brown markings in *M.bispinata*); and (3) pygofer with two medioventral processes not distinct (pygofer with three medioventral processes very distinct in *M.bispinata*).

## ﻿Discussion

Most members of the Tropidocephalini tribe with documented plant associations primarily feed on bamboo (Poales: Poaceae: Bambusoideae), as reported by Wilson et al. (1994), where this association accounts for 78% of the records. The remaining species are associated with various grasses (e.g., Wilson et al. 1994; Chen 2003; Chen and Tsai 2009; Qin and Zhang 2010). Specifically, species of *Malaxa* from China that have reported plant associations also feed on bamboo. Specimens have been collected from the leaves of several bamboo genera, including *Bambusa*, *Indocalamus*, *Fargesia*, and *Phyllostachys* (Yang and Yang 1986; Chen et al. 2006; Hou et al. 2013). We have also collected these two species of *Malaxa* from bamboo. These species may pose potential threats as pests in bamboo forests.

Based on geographic distribution (Fig. 1), all species of the genus *Malaxa* are found in the Oriental Realm (Holt et al. 2013), with a particularly rich diversity in China, where nine species are currently recorded. However, the genus is primarily distributed in Central China, South China, and Southwest China, with most species known only from their type localities. We believe that the actual distributions of many species remain unclear, as our findings are predominantly based on type localities, indicating a need for more extensive and in-depth investigations.

**Figure 27.**
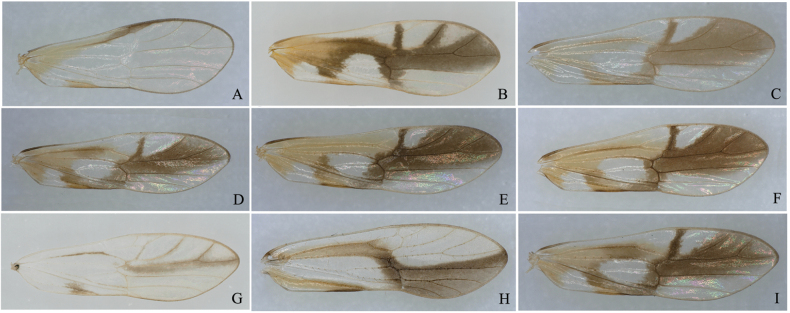
Forewings of Chinese *Malaxa* species **A***M.bispinata* Muir, 1926 **B***M.chongzuoensis* Lv & Chen, sp. nov. **C***M.delicata* Ding & Yang, 1986 **D***M.fusca* Yang & Yang, 1986 **E***M.hamuliferum* Li, Yang & Chen, 2019 **F***M.hunanensis* Chen, 2006 **G***M.longispina* Lv & Chen, sp. nov. **H***M.semifusca* Yang & Yang, 1986 **I***M.tricuspis* Li, Yang & Chen, 2019.

Bartlett and Kennedy (2018) redescribed the type species *M.acutipennis* Melichar, 1914. They observed several differences between *M.acutipennis* and the Chinese species in the genus *Malaxa*, the most salient difference is that *M.acutipennis* has an apically pointed forewing, while the forewings of all other *Malaxa* species are rounded. Here, we illustrate the forewings of all Chinese species of this genus, as shown in Fig. 27. We find that these forewings are also slightly different when extending towards the apex; some are relatively slender and apex slightly pointed, but the differences are not as pronounced as in the type species. Therefore, the study of this genus still requires more species, and it is highly necessary to expand the collection area for further collection.

## Supplementary Material

XML Treatment for Malaxa

XML Treatment for Malaxa chongzuoensis

XML Treatment for Malaxa longispina

## ﻿Additional information

### Author ORCIDs

Sha-Sha Lv https://orcid.org/0000-0001-5353-5082

Hong-Xing Li https://orcid.org/0000-0002-6427-8875

Lin Yang https://orcid.org/0000-0002-7841-5156

Yu-Bo Zhang https://orcid.org/0000-0002-6118-6190

Xiang-Sheng Chen https://orcid.org/0000-0001-9801-0343

### Data availability

All of the data that support the findings of this study are available in the main text.
